# Subsequent fracture risk after hip fracture surgery in China: a three-year retrospective cohort study

**DOI:** 10.1186/s12877-025-06871-z

**Published:** 2025-12-12

**Authors:** Yuan Yuan, Meng Zhang, Ping-Yang Li, Wei Wang, Yan-Na Lu, Ming-Hui Yang, Wei Tian, Jacqueline Close, Jing Zhang

**Affiliations:** 1 Department of Geriatrics, Beijing Jishuitan Hospital, Capital Medical University, Beijing, 100035 China; 2 National Center for Orthopaedics, Beijing, 100035 China; 3 Outpatient department, Beijing Jishuitan Hospital, Capital Medical University, Beijing, 100035 China; 4 Peking University Health Science Center, Beijing, 100191 China; 5 Department of Traumatic Orthopedics, Beijing Jishuitan Hospital, Capital Medical University, Beijing, 100035 China; 6 Neuroscience Research Australia, University of NSW, Sydney, NSW 2031 Australia; 7 School of Public Health, Harbin Medical University, Harbin, 150081 Heilongjiang China

**Keywords:** Hip fracture, Subsequent fractures, Risk factors, Osteoporosis, Follow-up

## Abstract

**Objective:**

This study aims to characterize subsequent fractures in a cohort of older adults undergoing hip fracture surgery in China, and to evaluate the risk factors associated with the subsequent fractures.

**Methods:**

In this retrospective cohort study, we collected 3-year postoperative follow-up data for older patients (aged 60 years and above) who were admitted and underwent surgery for hip fractures at a single center in Beijing, China, from 2016 to 2018. The patients were categorized into two groups: those who experienced a subsequent fracture during three years post-surgery and those who did not.

**Results:**

Within three years post-surgery, 63.7% (1,714/2,689) of patients had follow-up visits, with a cumulative incidence of subsequent fractures of 11.3% (193/1,714). Patients in the subsequent fracture group were more likely to be female (80.8% vs. 73.8%) and older (86.9 ± 6.4 vs. 85.1 ± 7.5) compared to the non-fracture group. They also had a lower BMI, higher rates of falls, and cognitive impairment or dementia. Both groups had a history of fragility fractures (16.6% [32/193] in the recurrent fracture group and 13.0% [198/1,521] in the non-recurrent fracture group), but osteoporosis was rarely recorded in medical records (3.7% and 0.6%, respectively). Cox regression revealed an inverted U-shaped age-risk relationship with an inflection point at 90 years: each year below 90 significantly increased refracture risk by 5.4% (HR 1.054 [95% CI: 1.013, 1.096]; *p* = 0.009), whereas above 90 showed a non-significant declining trend (HR 0.931 [95% CI: 0.855, 1.013]; *p* = 0.096). Dementia (HR 1.849 [95% CI: 1.209, 2.828]; *p* = 0.005), recent fall history (HR 1.552 [95% CI: 1.089, 2.210]; *p* = 0.015), and autumn-indexed fractures (HR 1.790 [95% CI: 1.119, 2.864]; *p* = 0.015) independently elevated refracture risk.

**Conclusion:**

This study assessed the status and long-term follow-up of subsequent fractures in hip fracture patients in China. Refracture risk is driven by nonlinear age dynamics (peaking at 90 years), modifiable predictors (dementia and recent falls), and seasonal vulnerability (autumn fractures). Implementing a fracture liaison service model tailored to national conditions is essential to reduce fragility fractures and subsequent fractures, especially for vulnerable populations.

**Supplementary Information:**

The online version contains supplementary material available at 10.1186/s12877-025-06871-z.

## Plain Language Summary

This study followed older adults after hip fracture surgery in China over three years, revealing an 11.3% subsequent fracture rate. Dementia, recent falls history, and autumn-indexed fractures independently elevated refracture risk. Implementing targeted interventions and fracture liaison services could help reduce secondary fractures, particularly in vulnerable populations.

**Supplementary Information:**

The online version contains supplementary material available at 10.1186/s12877-025-06871-z.

## Introduction

Hip fracture in older adults represents a significant health concern globally due to the associated high morbidity, mortality, loss of independence and healthcare costs. Hip fracture is also associated with a high risk of refracture and further associated increase in mortality rates. Over a 15-month period, Trevisan C et al. reported that one in every six patients admitted with a hip fracture (14.6%) was suffering a second hip fracture, and of those only 16.7% had been started on any antiresorptive therapy after the first hip fracture [1]. A recurrent hip fracture was also associated with poorer mobility (OR = 4.13, 95% CI: 1.23–13.84), higher rates of rehospitalization (OR = 2.57, 95% CI: 1.12–5.90), and mortality (HR = 1.81, 95% CI: 1.05–3.12), compared to first hip fractures [1].

A study from the China Health and Elderly Care Tracking Survey Database showed that the incidence of hip fractures among people over 45 years was 2.4% in 2015 [2]. Another study reported refracture rate was 25.6% among older people aged 60 years and above in China [3]. The management of hip fracture has been improved significantly in China, with a growing emphasis on multidisciplinary care models [4]. Additionally, identifying patients at high risk of subsequent fractures is a cost-effective secondary prevention strategy, benefiting both patients and the health care system. The Fracture Liaison Service (FLS) is a model of care to provide a systematic and multidisciplinary care pathway for postoperative patients with fragility fractures, through identifying patients with fragility fracture, investigating osteoporosis and risk of falls, and providing intervention of anti-osteoporosis and physical activity, which can significantly reduce refracture and mortality during recovery [5, 6]. It is noted that FLS has been implemented widely in high-income countries, but its adoption and implementation are limited and the status quo of post-surgery care of hip fracture remains unknown in China [7, 8], due to relatively marginalized primary health care facilities and underdeveloped continuity of post-surgery care in communities.

Given that a lack of evidence regarding the post-surgery care of older patients with hip fracture under a long-term follow-up in China, this study, therefore, leveraged detailed treatment data from those patients who were managed in a multidisciplinary ward to characterize 3-year refracture trajectories and then identify potentially modifiable risk factors.

## Methods

### Study design

This was a three-year retrospective cohort study from 2016 to 2018. We selected a 3-year follow-up period because it encompassed a critical window for exploring the impact of secondary fracture risk on long-term health outcomes of older patients with hip fracture, which will provide insights for better post-operative care management. Ethical approval was waived by Biomedical Ethical Committee of Beijing Jishuitan Hospital, Capital Medical University. Participants’ identifiable information were stored an independent server that cannot be accessed. Advanced Encryption Standard with 256-bit keys (AES-256) encryption was adopted for data protection, in compliance with national regulations on anonymized data research [9]. Informed consent was deemed unnecessary due to the retrospective and observational nature of the study (No. K2024-151–00).

### Study population and settings

The study population included patients with acute hip fracture admitted to the orthopaedic-geriatric co-management ward of a single center (Beijing Jishuitan Hospital, Capital Medical University) in Beijing, from January 2016 to December 2018. The inclusion criteria were: (1) age ≥ 60 years; (2) hip fracture confirmed by X-ray that occurred within the preceding 21 days; (3) underwent surgical repair of the fracture.

### Service configuration

A geriatrician-orthopaedics co-management ward was established in the hospital in 2015. In this care model, geriatricians are responsible for identifying osteoporosis, initiating anti-osteoporosis treatment, and offering consultations during follow-up. A discharge plan is developed to advise patients to return to hospital for regular follow-up visits at one month, six months, and one year after surgery.

### Data collection and outcomes

Data collected from hospital medical records included patients’ demographic characteristics, medical history, fracture types, clinical management details, and complications or comorbidities. Specifically, data on medical history were collected as follows: a history of cerebrovascular disease was confirmed by neuroimaging; a history of falls within the past year was documented from patient or caregiver reports alongside clinical records; and cognitive impairment was defined as a Mini-Mental State Examination (MMSE) score ≤ 26 at admission. Seasons of hip fracture were categorized according to meteorological seasons: Spring (March–May), Summer (June–August), Autumn (September–November), and Winter (December–February). Patients were followed for three years post-surgery and attended regular outpatient visits throughout this period. A geriatrician-led follow-up protocol was implemented to capture the occurrence and types of subsequent fractures, as well as to monitor the osteoporosis treatment adherence. All subsequent fractures documented in the medical records were identified using a comprehensive set of International Classification of Diseases, Tenth Revision (ICD-10) codes (see Supplementary Table 1). The primary outcome was the incidence of subsequent fractures. Secondary outcomes included a profile of osteoporosis treatments initiated in this high-risk population, and main risk factors of subsequent fracture.

### Statistical analysis

Patients were classified into two groups for analysis: those who sustained a further fracture during the study period and those who did not. Baseline characteristics were compared between the two groups using univariable analyses. Cox proportional hazards regression with sex stratification was employed for time-to-event analysis of refracture risk factors. The proportional hazards assumption was assessed using Schoenfeld residuals for each covariate; no violations were detected (all *p* > 0.05; see Supplementary Table 6). The multivariable model was adjusted for prespecified covariates, including demographics, comorbidities, and fracture-related variables. The model’s robustness was verified through checks of linearity, multicollinearity, and discriminative performance (adjusted C-statistic). Model calibration was evaluated using calibration plots. Missing data were addressed via multiple imputation by chained equations (MICE) with M = 20 imputations, including all outcome and predictor variables in the imputation model; pooled estimates were derived using Rubin’s rules. The Cox model assumes non-informative censoring. We assessed potential violation of this assumption by comparing baseline characteristics between retained and lost patients using standardized mean differences (SMDs), with an SMD > 0.1 defined as a significant imbalance.

## Results

### Study flow chart

The initial study sample comprised 2,689 patients. Of these, 15 (0.6%) patients died during hospitalization, and 960 (35.7%) were lost to follow-up after discharge. Ultimately, 1,714 (63.7%) retained in this whole cohort, contributing 4,812 person-years of observation.

**Fig. 1:**
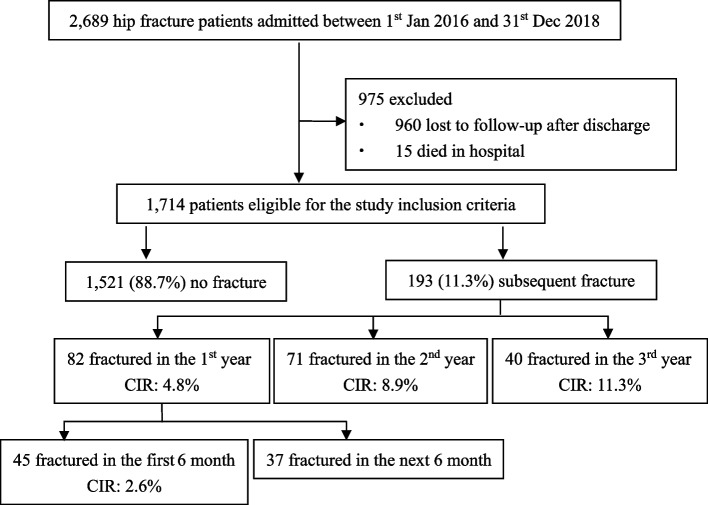
Flowchart of the study. (Post-discharge deaths were not adequately tracked throughout the entire follow-up period; CIR = cumulative incidence rate.)

**Fig. 2:**
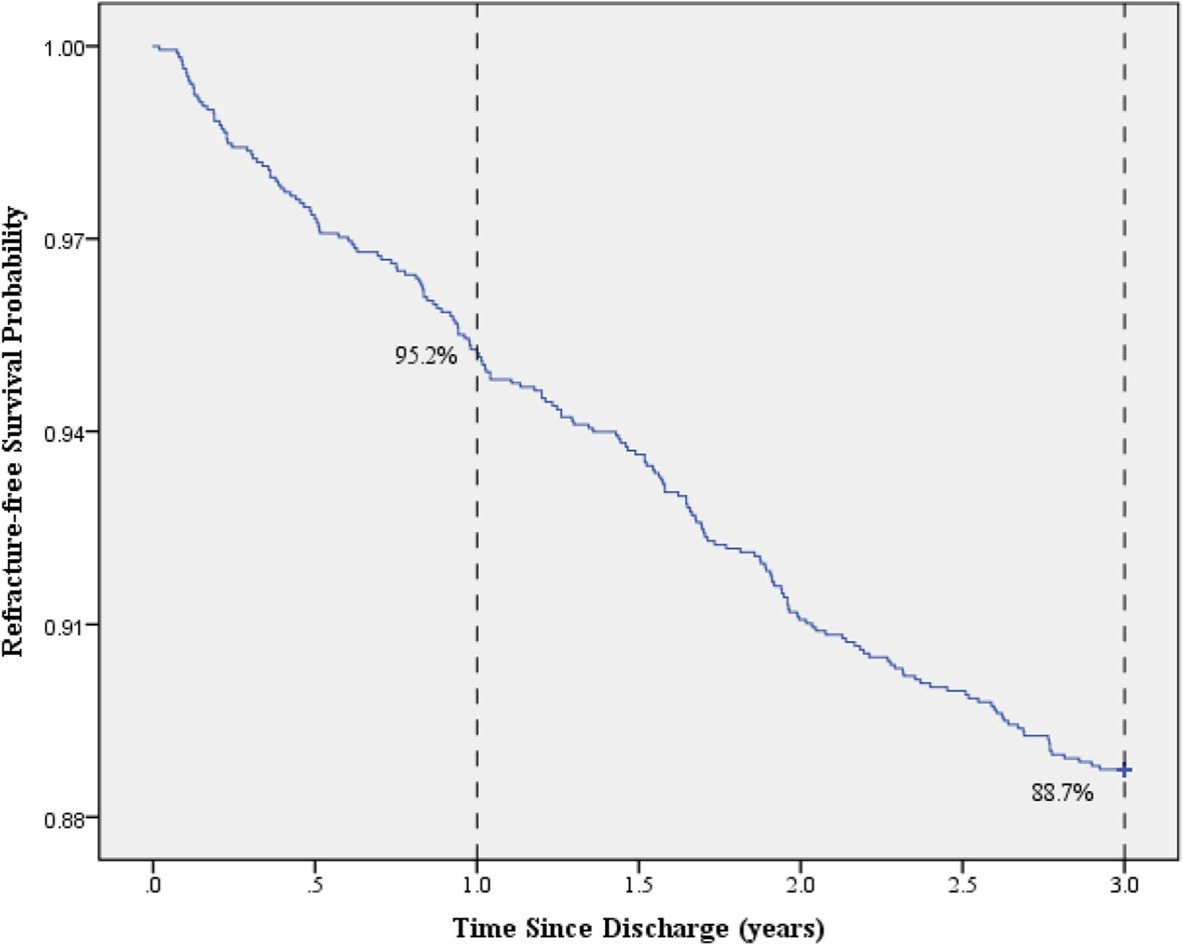
Kaplan–Meier Curve for Refracture-free Survival

Among the 1,714 patients, 1,521 (88.7%) did not experience subsequent fractures, while 193 (11.3%) sustained at least one further fracture. Of those with further fractures, 82 (42.5%) occurred within the first year, 71 (36.8%) in the second year, and 40 (20.7%) in the third year. Within the first year, 45 occurred in the first six months and 37 in the following six months (Figs. 1 and 2).

### Subsequent fracture sites

**Fig. 3:**
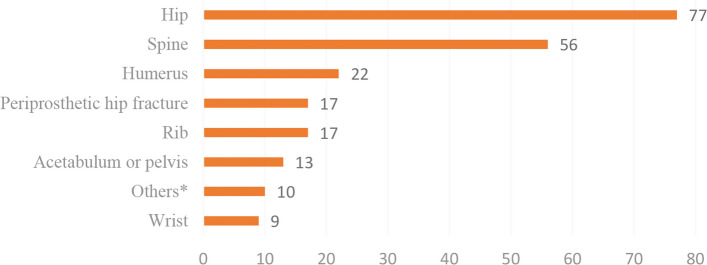
Numbers of subsequent fracture sites during the 3 follow-up years. (*Others include knee fractures 3, ankle fractures 2, patella fracture 1, metatarsal fracture 1, skull fracture 1, olecranon fracture 1, scapula fracture 1)

As depicted in Fig. 3, a total of 221 fractures occurred among which the 77 hip fracture patients were identified during the three-year follow-up. Four patients sustained three subsequent fractures, 20 sustained two, and 169 sustained one additional fracture. Following the ICD-10, the most common site for subsequent fractures was identified as the hip, followed by spine, humerus, periprosthetic hip, ribs, acetabulum or pelvis, and wrist, where more details were provided in Supplementary Table 1.

### Characteristics of subsequent fracture and non-fracture groups

Table 1 compared the characteristics of patients who did and did not sustain further fractures following hip fracture surgery. Patients in the subsequent fracture group were significantly more likely to be female (80.8% vs. 73.8%) and older (86.9 ± 6.4 vs. 85.1 ± 7.5), compared to the non-fracture group. Additionally, lower BMI, a history of falls, and cognitive impairment or dementia were significantly more prevalent in the subsequent fracture group.

**Table 1:** Characteristics of subsequent fracture and no-subsequent fracture groups among hip fracture patients and follow-up visits after surgery (*N* = 1,714)

**Variables**	**Subsequent fracture (*n* = 193)**	**No subsequent fracture (*n* = 1,521)**	χ^2^**/t/Z**	***P* Value**
Gender (Female)	156 (80.8%)	1,123 (73.8%)	4.426	*0.035*
Mean age ± SD	86.9 ± 6.4	85.1 ± 7.5	8.926	**<** *0.001*
Body Mass Index	21.9 ± 3.7	22.8 ± 3.8	0.045	*0.037*
Ever Smoking history	13 (6.8%)	118 (7.8%)	0.240	0.624
Ever Drinking history	5 (2.6%)	60 (3.9%)	0.845	0.358
History of falls in the past year	48 (24.9%)	252 (16.6%)	8.176	*0.004*
Number of falls M (IQR)	2 (1–2.8)	2 (1–3)	0.706	0.707
Type of hip fracture			4.373	0.118
Femoral neck fracture	101 (52.3%)	879 (57.8%)		
Intertrochanteric fracture	91 (47.2%)	616 (40.5%)		
Subtrochanteric fracture	1 (0.5%)	26 (1.7%)		
Season of hip fracture occurred			5.047	0.168
Spring	53 (27.5%)	383 (25.2%)		
Summer	52 (26.9%)	403 (26.5%)		
Autumn	60 (31.1%)	413 (27.2%)		
Winter	28 (14.5%)	322 (21.2%)		
Underlying medical conditions				
Hypertension	95 (49.2%)	800 (52.6%)	0.781	0.377
Insomnia	63 (32.8%)	441 (29.1%)	1.160	0.281
Diabetes	48 (24.9%)	397 (26.1%)	0.135	0.713
Coronary artery disease	38 (19.7%)	269 (17.7%)	0.468	0.494
Cerebrovascular disease	32 (16.6%)	213 (14%)	0.928	0.335
Dementia/Cognitive impairment	30 (15.5%)	118 (7.8%)	13.16	< *0.001*
Malignancy	7 (3.6%)	70 (4.6%)	0.380	0.538
Parkinson’s disease	5 (2.6%)	18 (1.2%)	2.562	0.109
Chronic respiratory disease	4 (2.1%)	50 (3.3%)	0.828	0.363
Anxiety or depression	3 (1.6%)	21 (1.4%)	0.037	0.847
Fractures prior to index hip fracture	32 (16.6%)	198 (13.0%)	1.871	0.171
Hip fracture	12 (37.5%)	80 (40.6%)		
Spine fracture	8 (25%)	44 (22.3%)		
Wrist fracture	1 (3.1%)	17 (8.6%)		
Patellar fracture	1 (3.1%)	12 (6.1%)		
Diagnosed osteoporosis in record (*n* = 188)	1 (3.7%)	1 (0.6%)	/	0.267 ^a^
Anti-osteoporosis	0	0	/	/
Calcium and/or Vit D	0	0	/	/
25OHD (ng/ml) (*n* = 1,330, M IQR)	11.9 (8.1–17.2)	11.8 (8.5–16.5)	−0.102	0.919
Osteocalcin (ng/ml) (*n* = 1,330, M IQR)	13.1 (9.4–17.9)	12.5 (9.0–18.9)	−0.676	0.499
Bone health treatment (Calcium and/or Vit D)	187 (96.9%)	1,477 (97.1%)	0.028	0.867
Anti-osteoporosis treatment	178 (92.2%)	1,388 (91.3%)	0.205	0.651
6 month follow-up after discharge	92 (47.7%)	1,066 (70.1%)	39.271	** < ***0.001*
Follow-up frequency in the first year M (IQR)	1 (0–2)	2 (1–3)	9.339	** < ***0.001*

*SD* standard deviation, *M (IQR)* median (interquartile range), *Vit D* vitamin D, *25OHD* 25-hydroxyvitamin D

^a^ Fisher’s exact test

Both groups had a history of fragility fractures (16.6% in the recurrent fracture group vs. 13.0% in the non-recurrent fracture group). However, the diagnosis rate of osteoporosis recorded in medical records was very low (3.7% vs. 0.6%), and none of the patients were receiving treatment for osteoporosis despite having previous low trauma fractures. Furthermore, the 6-month follow-up rate and the frequency of follow-up visits within the first year post-discharge were significantly lower in the subsequent fracture group (47.7% vs. 70.1%, *p* < 0.05; and 1 vs. 2, *p* < 0.05).

### Imbalance of participants retained and lost

Among patients who remained in the cohort and those lost to follow-up, substantial imbalances were observed in age (SMD = 0.38), dementia (SMD = 0.22), in-hospital bone health treatment (SMD = 0.33), and initiation of anti-osteoporosis treatment during hospitalization (SMD = 0.22). More details were shown in Supplementary Table 2. Given these observed significant imbalances, which indicated that the censoring assumption might be violated, we interpreted cautiously the hazard estimates. The baseline characteristics of the 15 deceased patients during the index hospitalization were presented as descriptive statistics stratified by sex in Supplementary Table 3.

### Investigation and treatment of bone health following hip fracture

There was no significant difference in 25OHD and osteocalcin levels between the two groups at admission. The use of anti-osteoporosis medication and Calcium/Vitamin D pre-fracture was absent (0%). Further, diagnosis (0.12%) and treatment (0%) of osteoporosis pre-admission were scarcely identified in their medical records. During hospitalization, 97% of participants received bone health treatment and 92% received anti-osteoporosis therapy; however, the proportion of those having oral intake at 1 year fell to 46% in both groups. Most patients received calcitonin treatment during hospitalization (91.2% vs. 88.6%). The usage rate of zoledronic acid was relatively low (16.1% vs. 18.2%), and the majority of patients received only one injection (77.4% vs. 84.5%). Patients with recurrent fractures more frequently used teriparatide (Supplementary Table 4).

### Cox proportional hazards regression analysis for risk factors of subsequent fracture

**Fig. 4:**
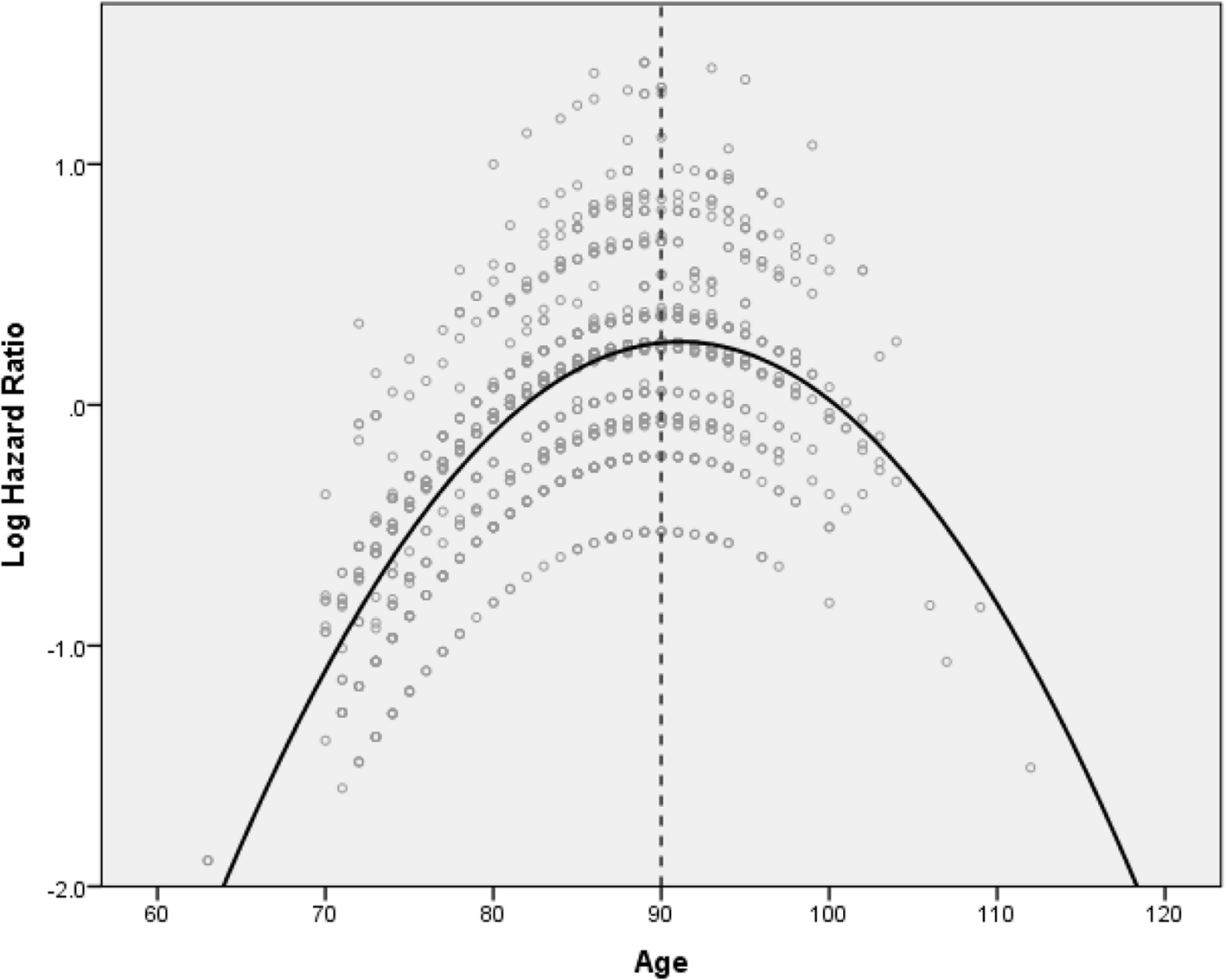
Nonlinear relationship between age and subsequent fracture risk

Cox proportional hazards regression identified several key predictors of subsequent fracture (Table 2). A significant inverted U-shaped relationship was observed between age and fracture risk, with an inflection point at 90.0 years (95% CI: 84.8–95.2). Age-stratified analysis (Table 3 and Fig. 4) revealed that patients under 90 years showed a 5.4% increased risk per additional year (HR 1.054 [95% CI: 1.013, 1.096]; *p* < 0.009), while those aged 90 or older exhibited a non-significant trend toward risk reduction (HR 0.931 [95% CI: 0.855, 1.013]; *p* = 0.096). Dementia (HR 1.849 [95% CI: 1.209, 2.828]; *p* = 0.005), history of falls (HR 1.552 [95% CI: 1.089, 2.210]; *p* = 0.015), and hip fracture in autumn (HR 1.790 [95% CI: 1.119, 2.864]; *p* = 0.015) were additional significant predictors of refracture. The modelling demonstrated modest discriminative ability (C-statistic = 0.627 [95% CI: 0.584, 0.671]). Detailed results of variable selection procedures, linearity testing, and multicollinearity assessment were provided in Supplementary Material (Model Performance and Validation).

**Table 2:** Final Cox model stratified by sex after backward elimination

Covariates		β	SE	Wald	HR	95% *CI*	*P* Value
Centered age		0.028	0.013	4.694	1.028	1.003–1.054	*0.030*
Centered age^2^		−0.003	0.001	4.571	0.997	0.994–1.000	*0.033*
Dementia/Cognitive impairment	No	Ref					
	Yes	0.615	0.217	8.048	1.849	1.209–2.828	*0.005*
History of falls in the past year	No	Ref					
	Yes	0.439	0.180	5.927	1.552	1.089–2.210	*0.015*
Season of hip fracture occurred	Winter	Ref					0.107
	Spring	0.450	0.243	3.427	1.569	0.974–2.527	0.064
	Summer	0.473	0.258	3.364	1.605	0.968–2.662	0.067
	Autumn	0.582	0.240	5.894	1.790	1.119–2.864	*0.015*

*SE* standard error, *HR* hazard ratio, *CI* confidence interval, *Ref.* reference

**Table 3:** Age-stratified fracture risk and interaction test

Group	n	HR per year	95% *CI*	*P* Value
< 90 years	1,212	1.054	1.013–1.096	0.009
≥ 90 years	502	0.931	0.855–1.013	0.096

*HR* hazard ratio, *CI* confidence interval, Interaction *p* = 0.011

### Subgroup analysis

Separate Cox models were constructed for secondary hip (*n* = 77) and non-hip fractures (*n* = 116). Advancing age, dementia, and autumn fracture significantly predicted secondary hip fractures. Subsequent non-hip fractures were associated with female sex, history of falls, dementia, and cerebrovascular disease. Dementia was a common risk factor across both fracture types. Full results are available in Supplementary Table 5.

### Sensitivity analysis

To evaluate whether early postoperative fractures disproportionately influenced risk associations, a sensitivity analysis excluding fractures within 90 days of index surgery was conducted (Supplementary Table 9). Associations for age (HR: 1.028 vs. 1.029), autumn (HR: 1.790 vs. 1.857) and dementia (HR: 1.849 vs. 1.798) retained magnitude and significance (*p* < 0.05). In contrast, history of falls transitioned from significant in the primary model (HR 1.552 [95% CI: 1.089, 2.210]; *p* = 0.015) to non-significant (HR 1.367 [95% CI: 0.917, 2.036]; *p* = 0.124), indicating its effect is concentrated within the immediate postoperative period where mobility impairment and rehabilitation challenges peak.

## Discussion

This study presented a 3-year cumulative refracture rate of 11.3% among older patients with hip fracture post-surgery. Age, history of falls, dementia or cognitive impairment prefracture, and autumn were identified as the major risk factors of refracture.

Compared with other studies, the 11.3% observed refracture is notably lower than global estimates (20–30%) [10]. This discrepancy could be due to the exclusion of cognitively impaired individuals, substantial loss to follow-up (36.3%), and deceased patients over the 3-year follow-up. Crucially, baseline disparities between retained and lost participants suggested that those lost were likely much older, more cognitively impaired, or lacked osteoporosis treatment. As a result, the non-random loss of higher-risk patients likely introduced selection bias, potentially underestimating the true refracture rate and limiting the generalizability of our findings to the broader fragility fracture population.

The low follow-up adherence underscored that there is a underdeveloped community healthcare system in China. Recent evidence indicated that China’s primary care faces a critical shortage of adequately trained general practitioners, which undermines a long-term follow-up care for complex geriatric recovery [11]. To address this, implementing integrated hospital-community continuous care models, such as nurse-led follow-up mechanism, supported by telemedicine and coupled with national investments in geriatric nursing, would reduce reliance on specialized manpower and establish sustainable long-term follow-up pathways.

It should be noted that although patients with subsequent fractures had lower 6-month follow-up rates and fewer annual visits, this correlation did not imply causation. The protective effect of attendance likely reflected reverse causality: healthier, more active patients are more likely to attend clinical visits. Furthermore, immortal time bias arose when defining exposure at 6 months, since patients must survive fracture-free for six months to qualify as attendees [12]. Therefore, follow-up attendance primarily reflects baseline health status rather than conferring protection.

Consistent with two studies in Canada, hip fractures constituted the most common subsequent fracture type in this cohort, accounting for 39.9% (77/193) of all refractures—a proportion slightly higher than the 33–34% reported in previous studies [13, 14]. This confirmed that an initial hip fracture significantly increases the risk of subsequent hip fractures, despite vertebral fractures being more common in general osteoporosis [15].

Our multivariable analysis yielded robust insights. While international studies had identified age, dementia, and other comorbidities as common risk factors, our analysis confirmed that and provided new perspectives [16–18]. Notably, we observed an inverted U-shaped relationship between age and refracture risk, with risk increasing until aged 90 before declining in the oldest-old, potentially due to survivorship bias, reduced mobility, or increased supervision in this age group. Furthermore, our study newly identified autumn as a significant seasonal risk factor. We hypothesized that patients suffering fracture in autumn are most susceptible during their rehabilitation phase, which coincides with the winter—a period of heightened environmental hazards—thus amplifying their risk of a refracture. Conversely, patients fracturing in winter may be partially shielded during this acutely vulnerable period due to supervised in-hospital care. These findings reinforced the well-established roles of dementia and falls history while introducing new considerations for age-specific and season-tailored prevention strategies.

Beyond risk factors, a critical care gap was observed in osteoporosis management. While osteoporosis affects 32.0% of Chinese adults aged 65 and above [19], the prefracture diagnosis rate was merely 0.1% in this cohort, without documented treatment, despite 13.2% of patients having a prior fracture history. This contrasted sharply with the 26.4% diagnosis and 46.2% treatment rates reported by United Kingdom Fracture Liaison Service Database (FLS-DB) annual report in 2017 [20]. Widespread Vitamin D deficiency was also evident (medians: 11.9 vs. 11.8 ng/mL), which may demonstrate insufficient attention to osteoporosis and suboptimal management of skeletal health prior to the fracture. While anti-osteoporotic treatment rates can reach over 90% during admission, calcitonin is the most medicine to use among patients, in order to relieve pain and reduce bone loss in the acute phase of hip fractures [21, 22]. Also, it is important for patients to receive bisphosphonates or denosumab postoperatively. Furthermore, the proportion of patients maintaining therapy at one year fell to approximately 46%, which underscores the severe disconnect between effective acute hospital care and long-term management in the community. These findings emphasized an urgent need to offer a comprehensive care through vertically integrating varied health sectors from tertiary hospitals to community health care centers. For instance, a primary prevention strategy regarding the identification of osteoporosis and the use of bone protection medication among high risk population should be implemented in community settings. A multidisciplinary team should be developed across different settings, adapted from FLS in UK, to provide a continuous, long-term rehabilitative strategy for older patients with hip fracture in China.

### Limitations

This study has several limitations. First, the exclusion of non-operatively managed patients that may have the frailest conditions with highest fracture risk and relatively low follow-up rate induced a selection bias, potentially underestimating the true subsequent fracture risk and affecting the generalizability of our evidence. Second, the absence of systematic post-discharge mortality data, combined with a lack of radiographic imaging and incomplete temporal documentation of the limited in-hospital deaths, may have an negative impact on the robustness of evidence. Third, we acknowledge inherent uncertainty regarding the formal statistical power to detect modest effect sizes or to conduct adequately powered subgroup analyses, particularly within strata with limited event counts. Lastly, generalizability is constrained by our urban tertiary-center cohort (median age 87 years, 97% in-hospital treatment), which diverges markedly from typical Chinese settings where hip fracture patients are younger and demonstrate lower treatment adherence.

## Conclusion

The study found that both patients with or without subsequent fractures had a lower follow-up rates after surgery. Despite a majority of patients being well-managed in hospital, the identification and primary prevention of osteoporosis were pretty underdeveloped. Age, dementia, history of falls, and autumn were identified as the main risk factors for subsequent fractures. These findings underscored that there is a need to not only establish a tailored Fracture Liaison Service model to reduce the incidence of subsequent fractures and provide secondary prevention strategies for older patients with hip fracture, but also develop a primary prevetion strategy, such as the identification of osteoporosis, in the community settings in China.

## Supplementary Information

Supplementary Material 1.

## Data Availability

The dataset is managed by Beijing Jishuitan Hospital, Capital Medical University. The data access request can contact the corresponding author.

## Abbreviations

25OHD: 25-Hydroxyvitamin D
AES-256: Advanced Encryption Standard with 256-bit keys
BMI: Body Mass Index
CI: Confidence Interval
CIR: Cumulative Incidence Rate
FLS: Fracture Liaison Service
FLS-DB: Fracture Liaison Service Database
HR: Hazard Ratio
ICD-10: International Classification of Diseases, Tenth Revision
M (IQR): Median (Interquartile Range)
MICE: Multiple Imputation by Chained Equations
MMSE: Mini-Mental State Examination
OR: Odds Ratio
Ref.: Reference
SD: Standard Deviation
SE: Standard Error
SMD: Standardized Mean Difference
Vit D: Vitamin D
