# Supplementary material for: Subsequent fracture risk after hip fracture surgery in China: a three-year retrospective cohort study
Source: BMC Geriatr. 2025 Dec 12;26:210. doi: 10.1186/s12877-025-06871-z (PMC12903599; doi:10.1186/s12877-025-06871-z)
Supplement: Supplementary file 1 — Supplementary Material 1. [file 12877_2025_6871_MOESM1_ESM.docx]

**Supplementary Methods**

**Study Groups and Sample Size Considerations**

Patients were classified into two groups for analysis: those who sustained a further fracture during the study period and those who did not. While a formal sample size calculation was not performed due to the retrospective design, our analysis adhered to the principle of ensuring an adequate number of events for stable model estimation. The final number of events (n = 193) comfortably exceeded the widely recommended minimum of 10–15 events per variable (EPV) for Cox proportional hazards regression, ensuring the robustness of our multivariable models.

**Comparison of Baseline Characteristics**

Baseline characteristics were compared between the two groups using appropriate univariable analyses. Continuous variables were first assessed for normality using the Shapiro-Wilk test. Normally distributed data are presented as mean ± standard deviation and were compared using Student’s*t*-test. Non-normally distributed data are presented as median (interquartile range) and were compared using the Mann-Whitney *U* test. Categorical variables, expressed as frequencies and percentages, were compared using the Chi-square test.

**Cox Regression Modeling Strategy**

Cox proportional hazards regression with sex stratification was employed for time-to-event analysis of refracture risk factors. The proportional hazards assumption was assessed using Schoenfeld residuals for each covariate; no violations were detected (all p > 0.05; see Supplementary Table 6). The multivariable analysis was constructed using prespecified clinical covariates based on biological plausibility and previous literature, including demographic factors, comorbidities, and fracture-related variables. A backward elimination approach using the Likelihood Ratio (LR) criterion was employed for variable selection, with a removal threshold of *p* > 0.10.

For continuous variables (e.g., age, 25OHD, osteocalcin), the assumption of linearity in the log-hazard was tested by including both the linear term and its quadratic term in initial multivariable-adjusted models. The improvement in model fit was assessed using likelihood ratio tests (LRT). A significant quadratic term (*p* < 0.05) indicated a violation of the linearity assumption.

For variables demonstrating significant non-linearity (age), the quadratic term was retained in the model. For all covariates, variance inflation factors (VIFs) were examined, with a VIF > 5 indicating severe collinearity and prompting further investigation. To mitigate the severe multicollinearity inherent between a variable and its polynomial term, the variable was centered at its mean value prior to creating the quadratic term. This process resolved multicollinearity, reducing all variance inflation factors (VIFs) to below 2.

**Model Performance and Validation**

The overall model performance was evaluated using the adjusted C-statistic (Harrell’s concordance index), which is analogous to the area under the ROC curve (AUC) for time-to-event data and provides a measure of the model’s discriminatory ability. Its 95% confidence intervals were also reported.

**Handling of Missing Data**

Missing data were addressed using multiple imputation by chained equations (MICE) with M=20 imputations, including all outcome and predictor variables in the imputation model. The results from these datasets were pooled according to Rubin’s rules to generate final estimates.

**Assessment of Potential Biases**

To evaluate potential selection bias introduced by loss-to-follow-up, we compared the baseline characteristics of patients who were retained in the final analysis versus those who were lost to follow-up. This comparison was made using standardized mean differences (SMDs), with an SMD > 0.10 indicating a meaningful imbalance between the groups.

**Supplementary Results**

**Assessment of Model Assumptions and Variable Selection**

Continuous variables (age, 25OHD, osteocalcin) were assessed for the assumption of linearity in the log-hazard. This was tested by including both the linear and quadratic terms of each variable in initial multivariable models. A significant nonlinear relationship was identified for age only (*p* < 0.05 for both the linear and quadratic terms), while 25OHD and osteocalcin exhibited linear relationships.

Severe multicollinearity was detected between the original age and age² terms (VIFs > 300). Centering age at the cohort mean (85.3 years) successfully resolved this issue, reducing all VIFs to acceptable levels below 2 (Supplementary Table 7).

The full prespecified model included the following covariates: demographic factors (centered age and centered age²), clinical risk predictors (dementia, Parkinson’s disease, cerebrovascular disease, 25OHD, osteocalcin, bone health treatment, and anti-osteoporosis treatment), and fracture-related variables (history of falls, prior fractures, and season of hip fracture). The covariate ‘gender’ was not tested for proportional hazards as it was handled through stratification. Backward elimination (Likelihood Ratio, removal criterion *p* > 0.10) was performed, which identified 5 retained predictors after 8 steps (Table 2 and Supplementary Table 8). The final model demonstrated modest discriminative ability (C-statistic = 0.627, 95% CI: 0.584–0.671).

**Nonlinear Age Effect and Stratified Analysis**

The final Cox model revealed a significant inverted U-shaped relationship between centered age and subsequent fracture risk. The inflection point—the age at which the risk trajectory reversed—was calculated using the formula derived from the model’s coefficients:

Inflection Point = |β₁ / (2 × β₂)| + Mean Age

Where β₁ is the coefficient for centered age (0.028) and β₂ is the coefficient for centered age² (-0.003), resulting in an inflection point of 90.0 years (95% CI: 84.8–95.2; calculated via bootstrap resampling).

Age-stratified analysis confirmed a significant divergence in fracture risk patterns across this inflection point (interaction *p* = 0.011). Among patients aged < 90 years (n = 1,212), each additional year of age increased fracture risk by 5.4% (HR 1.054, 95% CI: 1.013–1.096; *p* < 0.009). Conversely, in the ≥ 90 years cohort (n = 502), advancing age showed a non-significant trend toward risk reduction (HR 0.931, 95% CI: 0.855–1.013; *p* = 0.096).

**Predictors in the Final Multivariable Model**

Dementia significantly increased subsequent fracture hazard by 85.1% (HR 1.851, 95% CI: 1.210–2.831; *p* = 0.005), establishing it as a potent clinical predictor. A history of falls in the past year conferred a 55.3% higher risk (HR 1.553, 95% CI: 1.090–2.212; *p* = 0.015). A seasonal effect was also observed, with autumn hip fractures associated with a 78.8% elevated risk compared to winter fractures (HR 1.788, 95% CI: 1.118–2.861; *p* = 0.015). Female gender showed a non-significant trend toward increased risk (HR 1.368, 95% CI: 0.938–1.997; *p* = 0.104).

**Site-Specific Predictors of Subsequent Fracture**

To evaluate predictors for different fracture sites, separate multivariable Cox regression analyses were performed for subsequent hip fractures (n = 77) and non-hip fractures (n = 116).

For secondary hip fractures, dementia (HR 1.951, 95% CI: 1.040–3.660; *p* = 0.037), advancing age (per-year HR 1.037, 95% CI: 1.003–1.071; *p* = 0.031), and autumn season (HR 2.352, 95% CI: 1.137–4.864; *p* = 0.021) emerged as significant predictors, despite a borderline global season effect (*p* = 0.070).

Conversely, subsequent non-hip fractures were independently associated with female sex (HR 1.794, 95% CI: 1.032–3.118; *p* = 0.038), recent falls (HR 1.775, 95% CI: 1.119–2.815; *p* = 0.015), dementia (HR 1.892, 95% CI: 1.068–3.350; *p* = 0.029), and cerebrovascular disease (HR 1.758, 95% CI: 1.080–2.859; *p* = 0.023).

Notably, dementia was a significant predictor for both fracture types, while other predictors exhibited site-specific effects. Complete results are detailed in Supplementary Table 5.

**List of Supplementary Tables**

**Supplementary Table 1. Complete ICD-10 Codes with seventh-character extensions for subsequent fractures**

| **Fracture Type** | **ICD-10 Codes** |
| --- | --- |
| **Hip** | S72.0A, S72.1A, S72.2A |
| **Spine** | S12.0A, S12.1A, S12.2A, S12.7A, S22.0A, S22.1A, S32.0A, S32.702A, S32.712A, M48.5A |
| **Humerus** | S42.2A, S42.3A, S42.4A |
| **Periprosthetic hip** | M96.6A |
| **Ribs** | S22.3A, S22.4A |
| **Acetabulum or Pelvis** | S32.1A, S32.2A, S32.3A, S32.4A, S32.5A, S32.701A, S32.711A, S32.8A |
| **Wrist** | S52.5A, S52.6A, S62.0A, S62.1A |
| **Knee** | S72.4A, S82.1A |
| **Ankle** | S82.5A, S82.6A, S82.8A |
| **Patella** | S82.0A |
| **Metatarsal** | S92.3A |
| **Skull** | S02.0A, S02.1A |
| **Olecranon** | S52.0A |
| **Scapula** | S42.1A |

**Supplementary Table 2. Baseline characteristics of retained vs. lost patients**

| **Variables**  **N = 2,689 n (n%)** | **Retained (n = 1,714)** | **Lost (n = 975)** | **SMD** |
| --- | --- | --- | --- |
| **Gender (Female)** | 1,279 (74.6%) | 650 (66.7%) | 0.17 |
| **Mean age ± SD** | 85.3 ± 7.4 | 88.1 ± 7.1 | *0.38* |
| **Body Mass Index ± SD** | 22.7 ± 3.8 | 22.1 ± 4.0 | 0.15 |
| **Ever Smoking history** | 131 (7.7%) | 94 (9.7%) | 0.07 |
| **Ever Drinking history** | 65 (3.8%) | 48 (4.9%) | 0.05 |
| **History of falls in the past year** | 300 (17.5%) | 207 (21.2%) | 0.09 |
| **Type of hip fracture** |  |  |  |
| Femoral neck fracture (n = 1,435) | 980 (57.2%) | 455 (46.7%) | *0.21* |
| Intertrochanteric fracture (n = 1,207) | 707 (41.2%) | 500 (51.3%) | 0.20 |
| Subtrochanteric fracture (n = 47) | 27 (1.6%) | 20 (2.1%) | 0.04 |
| **Season of hip fracture** |  |  |  |
| Spring | 436 (25.4%) | 260 (26.7%) | 0.03 |
| Summer | 455 (26.5%) | 258 (26.5%) | 0 |
| Autumn | 473 (27.6%) | 259 (26.6%) | 0.02 |
| Winter | 350 (20.4%) | 198 (20.3%) | <0.01 |
| **Underlying medical conditions** |  |  |  |
| Hypertension | 895 (52.2%) | 533 (54.7%) | 0.05 |
| Insomnia (n = 2,682) | 504 (29.5%) | 264 (27.2%) | 0.05 |
| Diabetes | 445 (26.0%) | 25.7 (26.4%) | 0.01 |
| Coronary artery disease | 307 (17.9%) | 196 (20.1%) | 0.06 |
| Cerebrovascular disease | 245 (14.3%) | 146 (15.0%) | 0.02 |
| Dementia/Cognitive impairment | 148 (8.6%) | 152 (15.6%) | *0.22* |
| Malignancy | 77 (4.5%) | 43 (4.4%) | ＜0.01 |
| Parkinson’s disease | 23 (1.3%) | 19 (1.9%) | 0.05 |
| Chronic respiratory disease | 54 (3.2%) | 34 (3.5%) | 0.02 |
| Anxiety or depression | 24 (1.4%) | 9 (0.9%) | 0.05 |
| **Fractures prior to index hip fracture** | 230 (13.4%) | 121 (12.4%) | 0.03 |
| Hip fracture | 92 (40.2%) | 53 (43.8%) | 0.07 |
| Spine fracture | 52 (22.7%) | 20 (16.5%) | 0.16 |
| Wrist fracture | 18 (7.9%) | 16 (13.2%) | 0.17 |
| Patellar fracture | 13 (5.7%) | 3 (2.5%) | 0.16 |
| **25OHD ± SD (ng/ml, n = 2,038)** | 13.4 ± 7.5 | 12.1 ± 8.1 | 0.17 |
| **Osteocalcin ± SD (ng/ml, n = 2,038)** | 14.7 ± 8.3 | 14.9 ± 10.5 | 0.02 |
| **Bone health treatment (Calcium and/or Vit D)** | 1,664 (97.1%) | 870 (89.2%) | *0.33* |
| **Anti-osteoporosis treatment** | 1,566 (91.4%) | 822 (84.3%) | *0.22* |

SMD=Standardized mean difference; SMD interpretation: <0.10 = Negligible imbalance; 0.10-0.20 = Small imbalance; >0.20 = Substantial imbalance.

**Supplementary Table 3. Description of in-hospital mortality stratified by sex**

| **Variables N = 15 n (n%)** | **Male (n = 10)** | **Female (n = 5)** |
| --- | --- | --- |
| **Mean age ± SD** | 94.0 ± 5.2 | 89.6 ± 9.6 |
| **Ever Smoking history** | 2 (20.0%) | 1 (20.0%) |
| **Ever Drinking history** | 3 (30.0%) | 0 (0) |
| **History of falls in the past year** | 2 (20.0%) | 0 (0) |
| **Type of hip fracture** |  |  |
| Femoral neck fracture | 8 (80.0%) | 4 (80.0%) |
| Intertrochanteric fracture | 2 (20.0%) | 1 (20.0%) |
| **Season of hip fracture** |  |  |
| Spring | 3 (30.0%) | 0 (0) |
| Summer | 1 (10.0%) | 1 (20.0%) |
| Autumn | 2 (20.0%) | 3 (60.0%) |
| Winter | 4 (40.0%) | 1 (20.0%) |
| **Underlying medical conditions** |  |  |
| Hypertension | 7 (70.0%) | 3 (60.0%) |
| Insomnia | 2 (20.0%) | 3 (60.0%) |
| Diabetes | 5 (50.0%) | 0 (0) |
| Coronary artery disease | 3 (30.0%) | 1 (20.0%) |
| Cerebrovascular disease | 2 (20.0%) | 1 (20.0%) |
| Dementia/Cognitive impairment | 1 (10.0%) | 0 (0) |
| Malignancy | 0 (0) | 0 (0) |
| Parkinson’s disease | 1 (10.0%) | 0 (0) |
| Chronic respiratory disease | 0 (0) | 1 (20.0%) |
| Anxiety or depression | 0 (0) | 0 (0) |
| **Fractures prior to index hip fracture** | 3 (30.0%) | 0 (0) |
| **25OHD (ng/ml) (n = 9, M IQR)** | 7.7 (6.5–30.8) | 8.9 (7.1–11.2) |
| **Osteocalcin (ng/ml) (n = 9, M IQR)** | 7.7 (6.8–8.5) | 8.3 (6.3–19.1) |
| **Bone health treatment (Calcium and/or Vit D)** | 6 (60.0%) | 4 (80.0%) |
| **Anti-osteoporosis treatment** | 6 (60.0%) | 4 (80.0%) |

**Supplementary Table 4. Treatment of bone health following hip fracture**

| **Variables**  **N = 1,714 n (n%)** | **Subsequent fracture (n = 193)** | **No subsequent fracture (n = 1,521)** | χ² **/t** | ***P* Value** |
| --- | --- | --- | --- | --- |
| **Bone health treatment (Calcium and/or Vit D)** | | | |  |
| At discharge | 187 (96.9%) | 1,477 (97.1%) | 0.028 | 0.867 |
| Treatment duration > 1 yrs | 88 (47.1%) | 672 (45.5%) | 0.482 | 0.425 |
| **Anti-osteoporosis treatment** | |  |  |  |
| Calcitonin | 176 (91.2%) | 1,347 (88.6%) | 1.198 | 0.274 |
| Zoledronic acid | 31 (16.1%) | 277 (18.2%) | 0.537 | 0.464 |
| Injection once | 24 (77.4%) | 234 (84.5%) | 1.021 | 0.312 |
| Injection twice | 4 (12.9%) | 33 (11.9%) |  |  |
| Injection ≥ 3 times | 3 (9.7%) | 10 (2.9%) |  |  |
| Alendronic acid | 12 (6.2%) | 118 (7.8%) | 0.580 | 0.446 |
| Teriparatide | 12 (6.2%) | 49 (3.2%) | 4.479 | ***0.034*** |
| Raloxifene | 2 (1.0%) | 11 (0.7%) | 0.223 | 0.637 |
| VK2 | 0 (0) | 3 (0.2%) | 0.381 | 0.537 |

**Supplementary Table 5. Significant predictors from multivariable Cox regression analysis of site-specific subsequent fracture risk**

| **Covariates** | | **Subsequent hip fracture** | | | **Subsequent non-hip fracture** | | |
| --- | --- | --- | --- | --- | --- | --- | --- |
|  |  | **HR** | **95% *CI*** | ***P* Value** | **HR** | **95% *CI*** | ***P* Value** |
| Age | | 1.037 | 1.003–1.071 | 0.031 | / | / | / |
| Gender | Male | Ref. | | | | | |
|  | Female | / | / | / | 1.794 | 1.032–3.118 | 0.038 |
| History of falls in the past year | No | Ref. | | | | | |
|  | Yes | / | / | /. | 1.775 | 1.119–2.815 | 0.015 |
| Dementia/Cognitive impairment | No | Ref. | | | | | |
|  | Yes | 1.951 | 1.040–3.660 | 0.037 | 1.892 | 1.068–3.350 | 0.029 |
| Cerebrovascular disease | No | Ref. | | | | | |
|  | Yes | / | / | / | 1.758 | 1.080–2.859 | 0.023 |
| Season of hip fracture | Winter | Ref. | | | | | |
|  | Spring | 1.353 | 0.614–2.983 | 0.453 | / | / | / |
|  | Summer | 2.088 | 0.963–4.525 | 0.062 | / | / | / |
|  | Autumn | 2.352 | 1.137–4.864 | 0.021 | / | / | / |
|  | Global *P*-value for season | / | / | 0.070 | / | / | / |

HR=hazard ratio; CI=confidence interval; Ref.=reference.

**Supplementary Table 6. Assessment of the Proportional Hazards Assumption Using Correlation Analysis of Schoenfeld Residuals and Ranked Survival Time**

| **Covariate** | **Correlation with Ranked Time (r)** | ***P* Value** |
| --- | --- | --- |
| Centered age | -0.078 | 0.323 |
| Centered age^2^ | -0.049 | 0.534 |
| Dementia/Cognitive impairment | -0.145 | 0.064 |
| History of falls in the past year | -0.057 | 0.471 |
| Season of hip fracture (Spring vs. Winter) | 0.017 | 0.833 |
| Season of hip fracture (Summer vs. Winter) | -0.094 | 0.233 |
| Season of hip fracture (Autumn vs. Winter) | 0.021 | 0.792 |

The correlation coefficient ‘r’ represents the Pearson correlation between the Schoenfeld partial residuals for each covariate and the rank order of survival time. The covariate ‘gender’ was not tested for proportional hazards as it was handled through stratification.

**Supplementary Table 7. Variance inflation factor (VIF) comparison before and after age centering**

| **Varibles** | **Pre-centering VIF** | **Post-centering VIF** |
| --- | --- | --- |
| Age (original) | 323.992 | / |
| Age^2^ (original) | 323.819 | / |
| Centered age | / | 1.059 |
| Centered age^2^ | / | 1.010 |
| Gender | 1.052 | 1.052 |
| Dementia/Cognitive impairment | 1.036 | 1.036 |
| Parkinson’s disease | 1.013 | 1.013 |
| Cerebrovascular disease | 1.019 | 1.019 |
| 25OHD | 1.045 | 1.045 |
| Osteocalcin | 1.056 | 1.056 |
| Bone health treatment | 1.445 | 1.445 |
| Anti-osteoporosis treatment | 1.475 | 1.475 |
| History of falls in the past year | 1.034 | 1.034 |
| Fractures prior to index hip fracture | 1.024 | 1.024 |
| Season of hip fracture | 1.012 | 1.012 |

**Supplementary Table 8. Complete backward elimination steps (removal criterion: *p*>0.10)**

| **Step** | **Removed variable** | ***P* value at removal** | **Variables retained** |
| --- | --- | --- | --- |
| 1 | Osteocalcin | 0.952 | 11 |
| 2 | Anti-osteoporosis treatment | 0.604 | 10 |
| 3 | Bone health treatment | 0.584 | 9 |
| 4 | Fractures prior to index hip fracture | 0.362 | 8 |
| 5 | Parkinson’s disease | 0.285 | 7 |
| 6 | Cerebrovascular disease | 0.227 | 6 |
| 7 | 25OHD | 0.l57 | 5 |
| 8 | None (Final model) | / | 5 |

**Supplementary Table 9. Multivariable Cox regression analysis: primary vs. sensitivity analysis**

| **Covariates** | | **Original Model (n = 193)** | | | **Sensitivity Model (n = 166)** | | |
| --- | --- | --- | --- | --- | --- | --- | --- |
|  |  | **HR** | **95% *CI*** | ***P* Value** | **HR** | **95% *CI*** | ***P* Value** |
| Centered age | | 1.028 | 1.003–1.054 | 0.030 | 1.029 | 1.002–1.058 | 0.038 |
| Centered age^2^ | | 0.997 | 0.994–1.000 | 0.033 | 0.997 | 0.994–1.000 | 0.041 |
| History of falls in the past year | No | Ref. | | | | | |
|  | Yes | 1.552 | 1.089–2.210 | 0.015 | 1.367 | 0.917–2.036 | 0.124 |
| Dementia/Cognitive impairment | No | Ref. | | | | | |
|  | Yes | 1.849 | 1.209–2.828 | 0.005 | 1.798 | 1.123–2.878 | 0.015 |
| Season of hip fracture | Winter | Ref. | | | | | |
|  | Spring | 1.569 | 0.974–2.527 | 0.064 | 1.555 | 0.926–2.614 | 0.095 |
|  | Summer | 1.605 | 0.968–2.662 | 0.067 | 1.530 | 0.878–2.666 | 0.134 |
|  | Autumn | 1.790 | 1.119–2.864 | 0.015 | 1.857 | 1.116–3.089 | 0.017 |
|  | Global *P*-value for season | / | / | 0.107 | / | / | 0.127 |

HR=hazard ratio; CI=confidence interval; Ref.=reference.
